# Determination of the Properties of an Experimental Dental Composite with Addition of Hydroxyapatite with Varied Micro and Nano Particle Size: An Evaluation of Mechanical Properties, Ion Release, and Surface Characteristics †

**DOI:** 10.3390/jfb17080359

**Published:** 2026-07-26

**Authors:** Kacper Wiertelak-Makała, Joanna Nowak, Agata Szczesio-Włodarczyk, Piotr Wysocki, Aleksandra Zimon, Malgorzata Iwona Szynkowska-Jóźwik, Karolina Kopacz, Kinga Bociong

**Affiliations:** 1 Student Science Club “Materials in Dentistry”, Division of Dentistry, Faculty of Medicine, Medical University of Lodz, 251 Pomorska St., 92-213 Lodz, Poland; kacper.wiertelak-makala@student.umed.lodz.pl; 2 University Laboratory of Materials Research, Medical University of Lodz, 251 Pomorska St., 92-213 Lodz, Poland; joanna.nowak.1@umed.lodz.pl (J.N.); agata.szczesio@umed.lodz.pl (A.S.-W.); 3 Institute of General and Ecological Chemistry, Faculty of Chemistry, Lodz University of Technology, Zeromskiego 114, 90-543 Lodz, Poland; piotr.wysocki@p.lodz.pl (P.W.); aleksandra.zimon@p.lodz.pl (A.Z.); malgorzata.szynkowska@p.lodz.pl (M.I.S.-J.); 4 The Faculty of Medical Sciences, Warsaw Medical Academy, Rydygiera 8 St., 01-043 Warsaw, Poland; karolina.kopacz@umed.lodz.pl; 5 Department of Physiology, Pathophysiology and Clinical Immunology, Medical University of Lodz, Zeligowskiego 7/9 St., 90-752 Lodz, Poland; 6 Department of General Dentistry, Medical University of Lodz, 251 Pomorska St., 92-213 Lodz, Poland

**Keywords:** biomaterial, dental resin-based composite, bioactive filler, hydroxyapatite, mechanical properties, ion release

## Abstract

While resin-based dental composites (RBCs) are widely recognized as the golden standard in restorative dentistry, there is a need for improvement in their properties, including their potential biofunctional behavior. The aim of this study was to assess the impact of hydroxyapatite (HA) filler on the mechanical properties and provide preliminary evidence of biofunctional potential of prepared experimental RBCs. The effects of two forms of HA filler, mHA (2.5 μm) and nHA (<200 nm), in three concentrations (2.5, 5.0, and 7.5 wt.%) were tested in regard to hardness (HV), diametral tensile strength (DTS), flexural strength (TPB), flexural modulus, and calcium and phosphate ion release. Modification with HA improves the Vickers hardness and diametral tensile strength of RBCs, but has mixed effects on the flexural strength. The tested materials meet the minimum requirements for DTS and TPB for commercial composites, but their HV is closer to flowable materials. Calcium and phosphorus are detectable on the surface of HA-modified materials via SEM-EDS. There is also preliminary evidence of calcium and phosphate ion release in materials modified with HA, with the concentrations of 5 wt.% HA and above, providing the highest levels.

## 1. Introduction

### 1.1. Background

Since their introduction in the 1960s by Dr Rafael Bowen, resin-based dental composites (RBCs) have come to be acknowledged as the gold standard in restorative treatment of carious teeth due to their satisfactory mechanical properties and biocompatibility, easy application and workability, longevity, and pleasing aesthetics [1,2,3,4,5,6,7,8,9,10,11,12]. As composite materials, RBCs consist of at least two main components (phases): an organic resin matrix with a photoinitiator and an inorganic filler with silane as a bonding agent. Resin matrix is a mixture of methacrylate-based monomers capable of chain polymerization. Filler is predominantly composed of inorganic particles which improve the mechanical properties of RBC [1,2,3]. On average, filler makes up to 35–70 vol.% or 50–85 wt.% of the material [3,13,14]. Both phases have different properties, leading to the emergence of novel, improved properties in the composite material. Functional filler can be added to improve overall performance and introduce new functions to RBC.

Contemporary dentistry prioritizes the conservation of as much tooth structure as possible [7,15]. The risk of secondary caries is a major challenge of RBC restorations. This process results from biofilm stagnation at the surface of RBCs and microleakage, leading to changes in local chemical environment and degradation of bonded interface as well as demineralization of tissues [1,2,16,17,18,19,20]. Degradation of interface and demineralization of dental tissue results in treatment failure with annual rates of 3–11%, requiring restoration replacement [1,2]. Thus, there is a need for development of bioactive RBCs that limit the risk of secondary caries by providing calcium and phosphate release for remineralization. There has been substantial focus on research on restorative materials with regenerative potential in recent years [21].

World Dental Federation (FDI) in 2022 issued a policy statement that requires bioactive dental restorative materials to meet all of the following criteria:

- Clearly defined and described mechanism (biological, mixed or chemical);
- Scientifically proven bioactive effect in vitro or in situ and preferably in clinical studies;
- Stated duration of effect;
- No significant adverse biological side effects;
- The prime purpose, e.g., to be used to rebuild the form and function of lost tooth substance is not impaired as demonstrated by data from in vitro and clinical studies [22,23].

Examples of bioactive chemical mechanisms include remineralization of hard dental tissues due to calcium and phosphate ion release [7,15,24,25,26,27,28]. Regarding the materials’ primary function, their mechanical properties must not be impaired. ISO 4049 includes one standardized mechanical test—the three-point bending test for flexural strength—but hardness, diametral tensile strength, and contraction stress are typically tested due to their influence on the clinical performance of RBC [17,29]. The FDI policy statement provides a comprehensive evaluation framework for bioactivity. This framework should be aspirational for all studies in the field. In the context of this preliminary study, the authors decided to focus on researching whether the primary purpose is not impaired. Additionally, preliminary evidence of potential biofunctional behavior and its mechanism are explored with respect to calcium and phosphate ion release.

Hydroxyapatite (HA)—Ca_10_(PO_4_)_6_(OH)_2_—has been proposed as a promising functional filler for bioactive RBCs [1,3,4,6,7,25,30,31,32,33,34]. HA is a major component of bone and hard dental tissues characterized by high biocompatibility and biofunctional potential [1,3,5,19,30,32,35,36]. HA is able to release calcium (Ca^2+^) and phosphate (PO_4_^3−^) ions, promoting remineralization in simulated environment [1,4,6,16,19]. HA filler can form a precipitate layer of those ions on RBC surface immersed in artificial saliva [1,37]. HA is available in a wide range of isomorphic ion substitutions and in multiple forms, such as whiskers, spheres, fibers, and rods from the micro to the nanoscale [4,6,38].

### 1.2. Aim of the Study

RBCs require further improvements. HA is a promising functional filler with the potential to enable remineralization via ion release and HA layer formation on the surface of the material. However, HA’s impact on RBCs’ mechanical properties needs to be studied before assessing its potential biofunctional behavior. Biofunctional potential via ion release would be a considerable improvement for HA-modified materials, but their primary function must not be impaired. Thus, the aim of this study is to characterize the mechanical properties of HA-modified materials and to provide preliminary evidence of potential biofunctional behavior, which might lead to further studies designed to fully explore bioactive potential of HA-modified materials in accordance to FDI framework presented in Section 1.1.

The authors posed the following PICO question: In RBCs, what is the impact of HA as a functional filler compared to RBCs without HA on the mechanical performance and biofunctional potential of the material?

The null hypothesis was that there would be no significant difference between the properties of HA-modified and unmodified RBCs. The alternative hypothesis was that there would be an improvement in tested qualities for HA-modified RBCs.

## 2. Materials and Methods

### 2.1. Composition of Experimental Materials and Sample Preparation

Resin matrix: all materials shared the matrix with monomers bisphenol-A-glycerolatedimethacrylate (Bis-GMA, purity 98%), diurethane dimethacrylate (UDMA, purity 97%), triethylene glycol dimethacrylate (TEGDMA, purity 95%) and 2-hydroxyethyl methacrylate (HEMA, purity 97%) (all from Sigma-Aldrich Co., Ltd., St. Louis, MO, USA). The appropriate amounts of each monomer were added to the mixture in a weight ratio of 35/35/20/10 wt.%, respectively. The mixture was placed in an opaque glass container, heated on a hotplate at 60 °C to increase the mobility of monomer chains and mixed manually with a glass rod until a clear, homogenous solution was obtained. After preliminary homogenization, the polymerization stabilizer butylated hydroxytoluene (BHT) and conventional camphoroquinone-amine initiator system with camphoroquinone (CQ) as initiator and 2-(dimethylamine)ethyl methacrylate (DMAEMA) (all from Sigma-Aldrich Co., Ltd., St. Louis, MO, USA) as co-initiator were added in weight ratio of 0.1/0.4/0.9 wt.%, respectively. The mixture was mixed with a magnetic stirrer until a homogenous solution was obtained. The resin matrix was stored in the dark to limit light exposure and avoid unintentional polymerization [39]. Silica filler: silica Arsil (ZakładyChemiczneRudniki S.A., Rudniki, Poland) was silanized with γ-methacryloxypropyltrimethoxysilane (γ-MPTS) purchased from Unisil (Tarnów, Poland) following the protocol by Kleczewska et al. [40]. Hydroxyapatite filler: HA powder with particle size of 2.5 μm (mHA) and HA nanopowder with particle size < 200 nm (nHA) was purchased from Sigma-Aldrich Co., Ltd., St. Louis, MO, USA. Additionally, a hybrid mixture of both forms of HA in equal parts was prepared (hHA).

Eight groups of experimental materials were prepared (Table 1). Unmodified material with 45 wt.% silica and 55 wt.% resin matrix served as negative control. Constant silica concentration was designed to provide satisfactory balance of manufacturing feasibility and mechanical properties, as demonstrated in previous laboratory experience [13]. The proposed composition of experimental materials is presented in the table below.

In total, 6 g of each material was prepared as above. An appropriate amount of resin matrix was placed in a plastic container and mixed with HA using HauschildSpeedMixer (TM DAC 150 FVZ, Hauschild and Co., Ltd., Hamm, Germany) at 1500 rpm for 30 s. Small portions of silica (0.15–0.20 g) were added progressively to ensure equal distribution of the filler in the matrix and mixed automatically at 1500–3000 rpm for 30 s after each portion, with speed dependent on the mixture viscosity. The increase in mixing cycles was dependent on the increasing mixture viscosity. Once complete filler load was introduced, the composite was mixed automatically three times at 3000 rpm for 60 s. The materials were then ground manually in a mortar for 10 min. This hybrid mixing method was chosen because previous laboratory experience indicates mechanical mixing improves hardness and flexural strength of experimental RBCs, while hybrid manual mixing yields lower contraction stress [41]. Containers were isolated from light.

Prepared materials were divided into samples by placing them in silicon molds between two microscopic slides and polyester stripes to avoid the formation of an inhibition layer. Samples were cured for 20 s per 1.5 mm at 1200 mW/cm^2^ using CURE TC-1 polymerization lamp (Spring Health Products, Norristown, PA, USA) with direct contact of the optical fiber with the microscopic slide surface. Polymerization was carried out on both sides of each sample. Before polymerization, the light curing lamp was inspected with a radiometer system (Digital Light Meter 2000, Rolence Enterprise Inc., Taoyuan, Taiwan).

### 2.2. Hardness

Hardness of the experimental materials was tested using the Vickers method (HV). The semi-automatic Zwick ZHV2 hardness tester (Zwick-Roell, Ulm, Germany) was used in this procedure. The specimens were placed perpendicularly to the indenter [42]. The Vickers diamond indenter in the shape of a pyramid with a normal load of 1000 g (9.81 N) was applied with a contact time of 10 s to cylindrical samples with a height of 3 mm and a diameter of 6 mm. Nine measurements were performed for each group (*n* = 9) with three indentations per sample. All measurements were performed by the same researcher to achieve acceptable reproducibility.

### 2.3. Diametral Tensile Strength

Diametral tensile strength (DTS) was tested on a universal testing machine Zwick-Roell Z020 (Zwick-Roell, Ulm, Germany) according to diametral tensile test (Brazilian test) protocol, frequently used for dental composites and other brittle materials [42]. Axial loading at a constant crosshead speed of 2 mm/min was applied until the maximum force destroyed the cylindrical sample with the dimensions used in the HV test. The sample rested on the side, allowing the upper and lower platens of the testing machine to apply force to a line axis across the entire length of the specimen [42]. Eleven specimens were used for each study group (*n* = 11). The formula describing the results is as follows:

$$DTS=\frac{2F}{\pi DT}\;[\mathrm{MPa}]$$

where:

*F*—compressive force that destroys the sample [N];
*D*—diameter of the specimen [mm];
*T*—thickness of the specimen [mm].

### 2.4. Three-Point Bending Test

Flexural strength (FS) was tested by the standard ISO 4049:2019 protocol for the three-point bending test (TPB) for dental composites, a typical procedure for flexure testing [29,42]. A universal testing machine Zwick-Roell Z020 (Zwick-Roell, Ulm, Germany) was used. Constant cross-head speed of 1 mm/min was applied to rectangular beam-shaped samples with the dimensions of 25 mm × 2 mm × 2 mm at a support spacing of 20 mm until the maximum force destroyed the sample. Seven specimens were used for each group (*n* = 7). The results were returned automatically. The formula describing the test results is as follows:

$$TPB=\frac{3FL}{2bh^{2}}\;[\mathrm{MPa}]$$

where:

*L*—support spacing, 20 [mm];
*F*—load [N];
*b*—width of the specimen [mm];
*h*—height of the specimen [mm].

Additionally, the application of the three-point bending test allowed for the analysis of flexural modulus.

### 2.5. Inductively Coupled Plasma Optical Emission Spectrometry of Ion Release

Cylindrical samples as described above were prepared. There was no surface-area-to-volume ratio standardization. Samples were stored in plastic containers in ultrapure water (50 mL) for 1, 2, 3, and 4 weeks. No pH controls were included. Three specimens were prepared for each time period and stored individually (*n* = 3). Reported values had an interval character. Each specimen was immersed for only one assigned period and measured once, rather than being followed longitudinally. Inductively coupled plasma optical emission spectrometry (ICP-OES) was used for the quantitative analysis of calcium (Ca) and phosphate (P) ion release using iCAP 7400 Series apparatus (ThermoScientific, Waltham, MA, USA). The limit of quantification (LOQ) for calcium (393.366 nm) was 0.009 mg/L and LOQ for phosphates (177.495 nm) was 0.005 mg/L.

### 2.6. Scanning Electron Microscopy with Energy-Dispersive X-Ray Spectroscopy

The surface morphology and elemental composition of the samples were examined using scanning electron microscopy (SEM, Hitachi S-4700, Tokyo, Japan) equipped with energy-dispersive X-ray spectroscopy (EDS, Thermo Scientific UltraDry, Madison, WI, USA). Prior to analysis, the specimens were sputter-coated with either gold or carbon to improve surface conductivity. The coatings were deposited using a 208HR sputter coater (gold) or a 108 Carbon/A coater (carbon) (Cressington Scientific Instruments Ltd., Watford, UK). SEM imaging was performed at an accelerating voltage of 25 kV. Secondary electron (SE) images were acquired at different magnifications (1000×, 5000×, 10,000×, and 20,000×). Elemental distribution maps and EDS spectra were acquired at a magnification of 1000×.

### 2.7. Statistical Analysis

The statistical analysis was conducted using the Shapiro–Wilk test to assess the normality of the data distribution. Given that the data deviated from a normal distribution, the Kruskal–Wallis test was employed to evaluate differences among independent groups. When significant differences were detected, posthoc pairwise comparisons were performed using Dunn’s multiple comparison test. To control for type I error associated with multiple testing, Bonferroni correction was applied. All statistical analyses were performed using Statistica ver.13.3 (Tibco Software Inc., Krakow, Poland). The level of statistical significance was set at *p* < 0.05.

## 3. Results

### 3.1. Composition of Experimental Materials and Sample Preparation

It should be noted that the introduction of full filler content was becoming increasingly difficult as the overall filler load increased, and the materials were becoming more brittle. While this observation is purely qualitative, its potential influence on the material’s rheological properties should be considered. This effect was more noticeable for nHA. While the designed silica content for all materials was to be 45.0 wt.%, materials modified with 7.5 mHA and 7.5 nHA presented with a shortfall of 1.5 and 2.2 wt.%, respectively. Thus, the obtained concentrations are presented in Table 2.

### 3.2. Hardness

**Figure 1:**
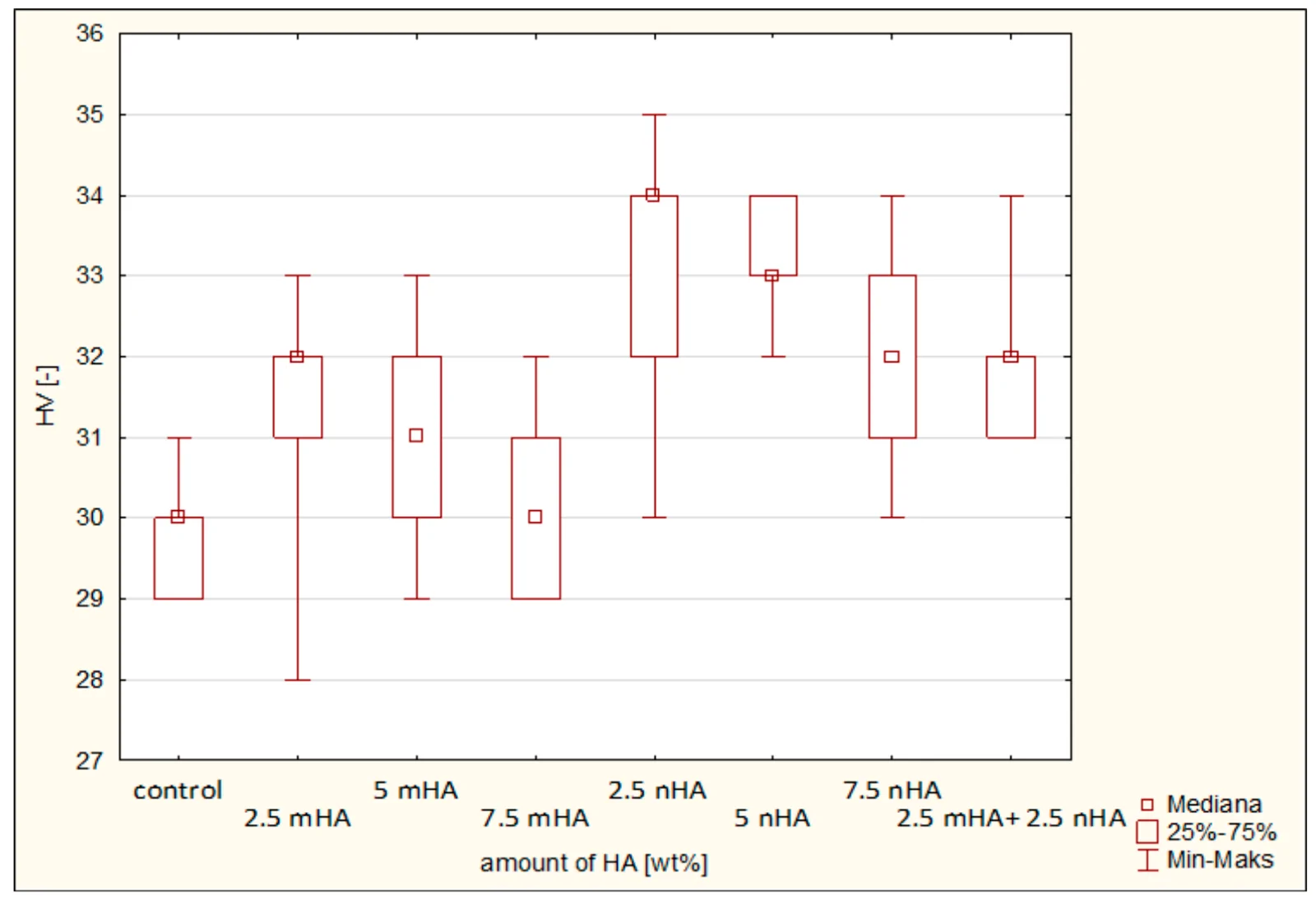
Vickers hardness (*n* = 9) of experimental materials.

The highest median HV value was observed in RBC modified with 2.5 wt.% nHA. The obtained results are presented in Table A1. Low coefficient of variation (CV) values could indicate very good precision and repeatability of the method. However, there was an inconsistency with the normal distribution of data. The differences in HV between the study groups were statistically significant (*p* = 0.000, Kruskal–Wallis test). Notably, there was a statistically significant increase in HV observed in RBC modified with 2.5 (*p* = 0.0012) and 5 wt.% nHA (*p* = 0.0001) in comparison to the control. Statistically significant differences were also observed between RBC modified with 2.5 wt.% nHA (*p* = 0.0305) and 5 wt.% nHA (*p* = 0.0030) and material modified with 7.5 wt.% mHA, with both materials modified with nHA exhibiting increased HV in comparison to mHA. The results are presented in Figure 1.

### 3.3. Diametral Tensile Strength

**Figure 2:**
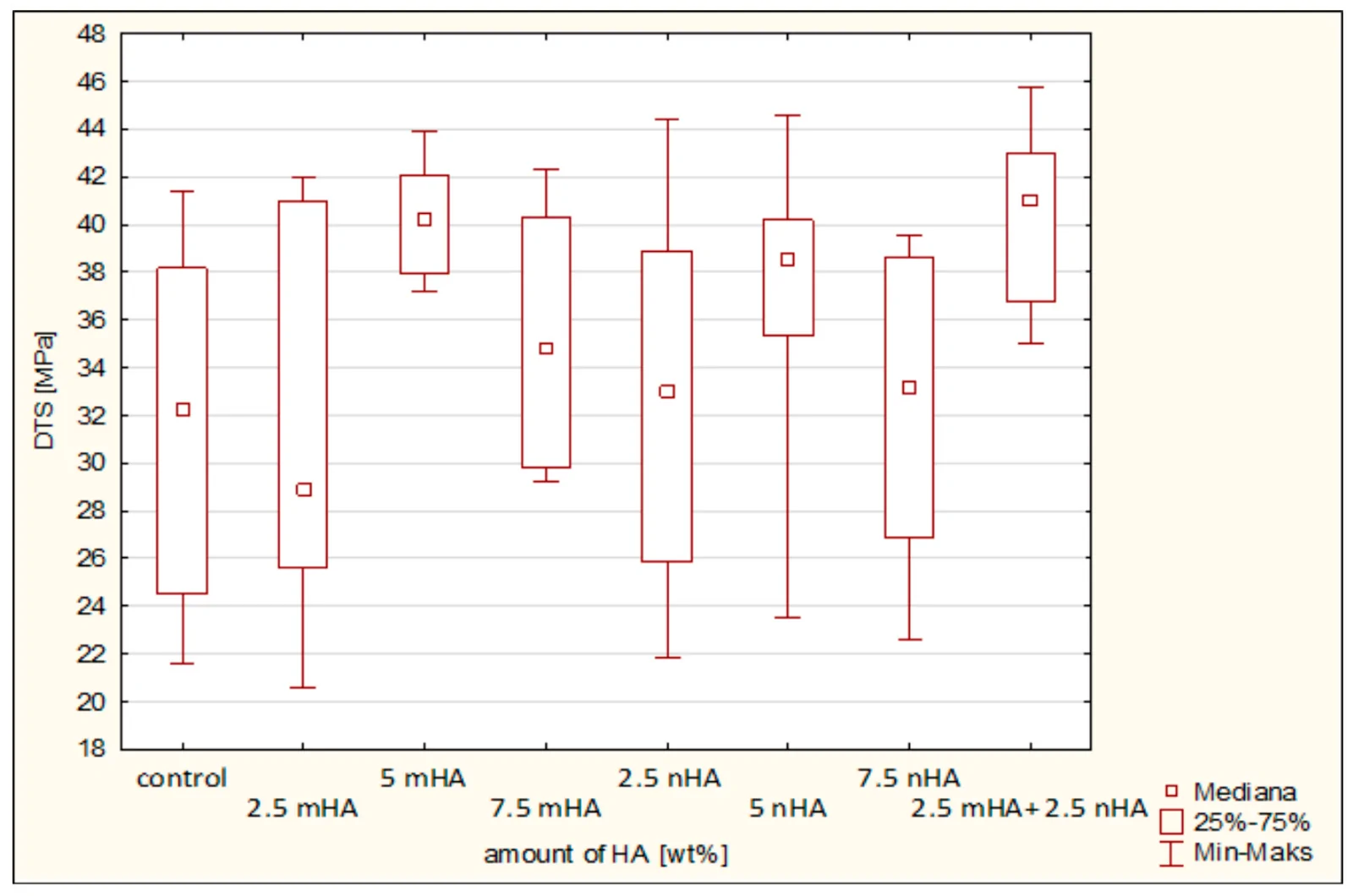
Diametral tensile strength (*n* = 11) of experimental materials.

The highest median DTS value was observed in 5 wt.% hHA-modified composite. The results are presented in Table A2. Similarly to HV, low CV values could indicate good repeatability of the method. The results were inconsistent with normal distribution. The differences in DTS between groups were statistically significant (*p* = 0.0022, Kruskal–Wallis test). There was a noteworthy statistically significant increase observed for RBC modified with hybrid mixture of 2.5 wt.% mHA and 2.5 wt.% nHA—5 wt.% hHA (*p* = 0.0350) in comparison to the control. The results are presented in Figure 2.

### 3.4. Flexural Strength

**Figure 3:**
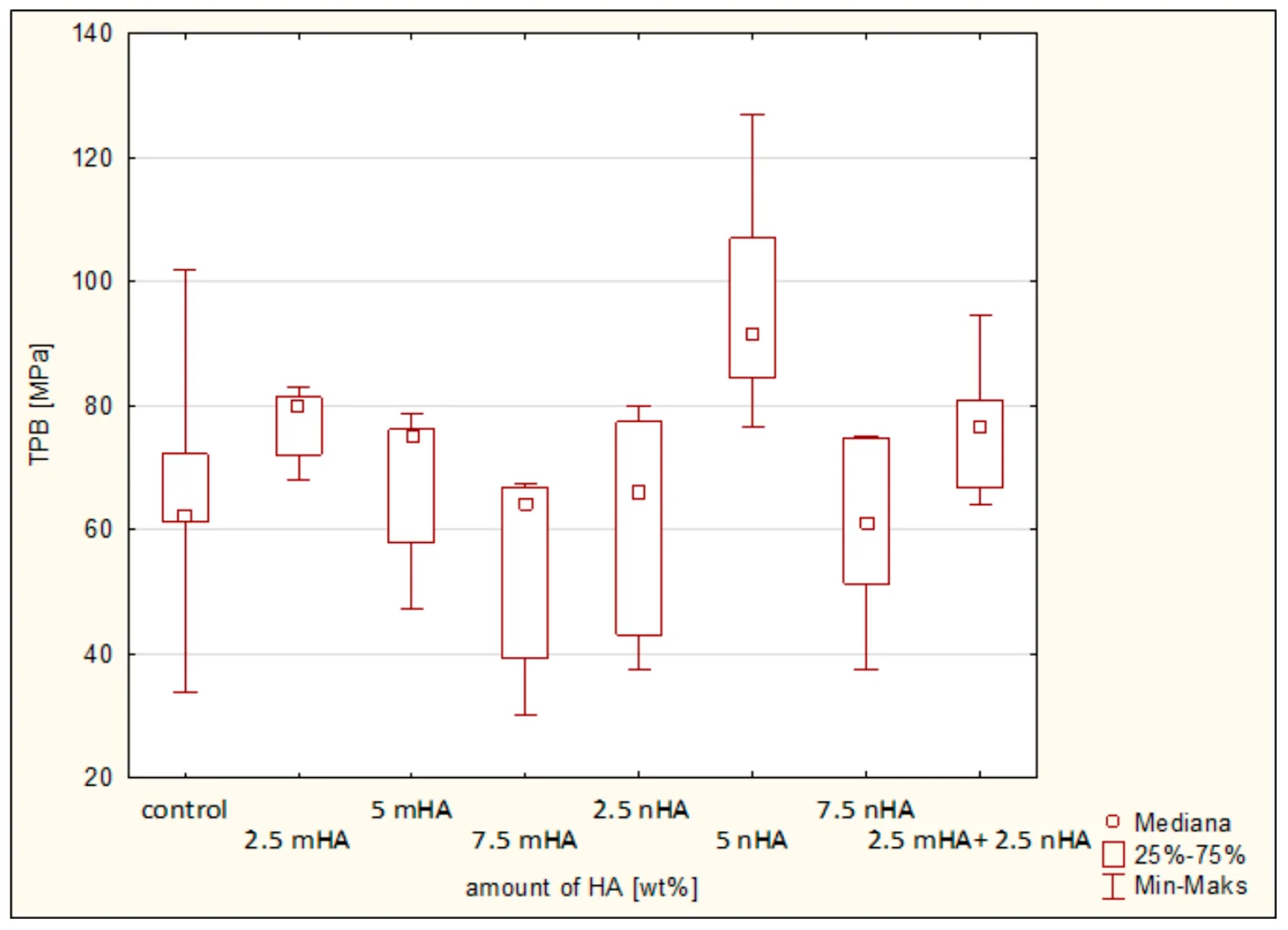
Flexural strength (*n* = 7) of experimental materials.

The highest median TPB values were observed in RBCs modified with 5 wt.% nHA. The results are presented in Table A3. Low CV values could indicate acceptable repeatability of the method. However, the data did not follow a normal distribution. The differences in TPB between groups were statistically significant (*p* = 0.004, Kruskal–Wallis test). RBC modified with 5 wt.% nHA exhibited a statistically significant (*p* = 0.0369) increase in TPB in comparison to the control. Statistically significant differences were also observed for RBC modified with 5 wt.% nHA and 2.5 wt.% nHA (*p* = 0.0391), 7.5 wt.% nHA (*p* = 0.0060) and 7.5 wt.% mHA (*p* = 0.0016), with 5 wt.% nHA exhibiting superior TPB values over those materials. The results are presented in Figure 3.

### 3.5. Flexural Modulus

**Figure 4:**
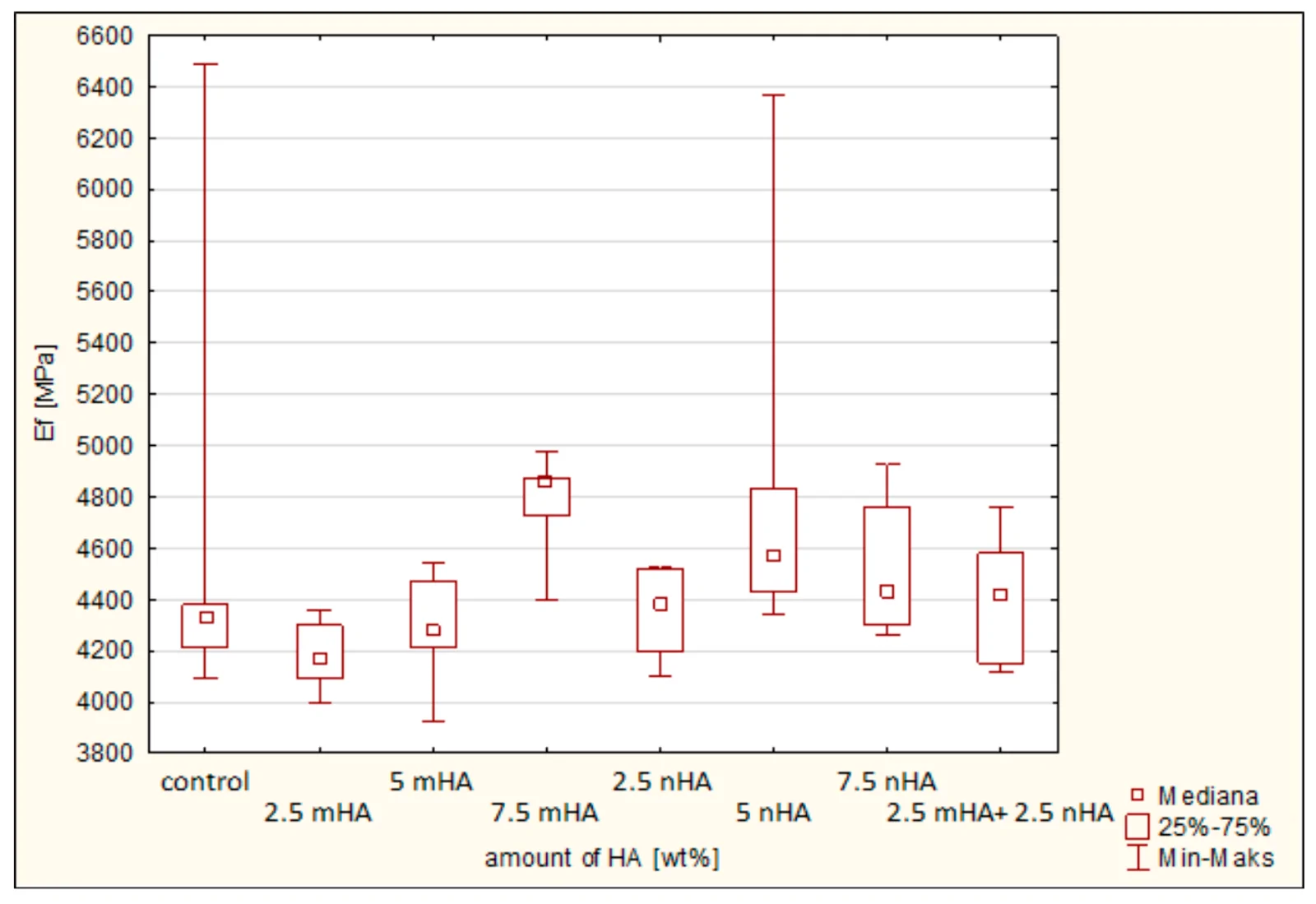
Flexural modulus (*n* = 7) of experimental materials.

The highest median Ef value was observed in RBC modified with 7.5 wt.% mHA. The results are presented in Table A4. Low CV values could indicate very good repeatability of the method. The results were inconsistent with a normal distribution. The differences in Ef between groups were statistically significant (*p* = 0.0017, Kruskal–Wallis test). There was a statistically significant increase in flexural modulus observed for materials modified with 7.5 wt.% mHA (*p* = 0.0022) and 5 wt.% nHA (*p* = 0.0402) in comparison to RBC modified with 2.5 wt.% mHA. The results are presented in Figure 4.

### 3.6. Inductively Coupled Plasma Optical Emission Spectrometry of Ion Release

**Figure 5:**
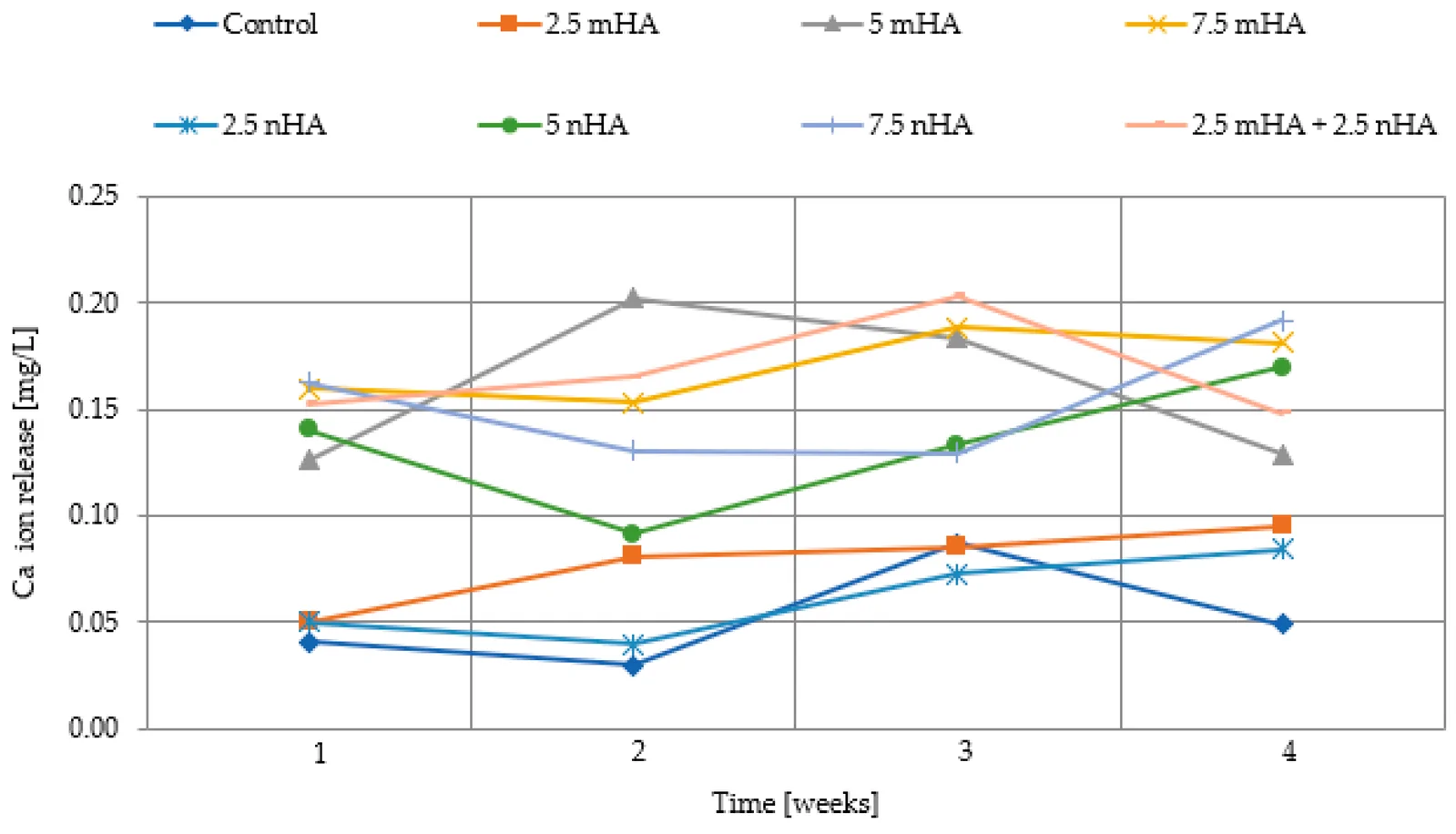
Dynamic of calcium (Ca) (393.366 nm) ion release via ICP-OES.

Calcium ion release is presented in Figure 5 and Table A5. At virtually all HA concentrations and time points, modified materials released more calcium ions than the control. The effect of calcium ion release is dose-dependent and sustained over the period of 4 weeks. The 5 wt.% hHA formulation provided the highest Ca ion release at 0.20 mg/L after 3 weeks.

**Figure 6:**
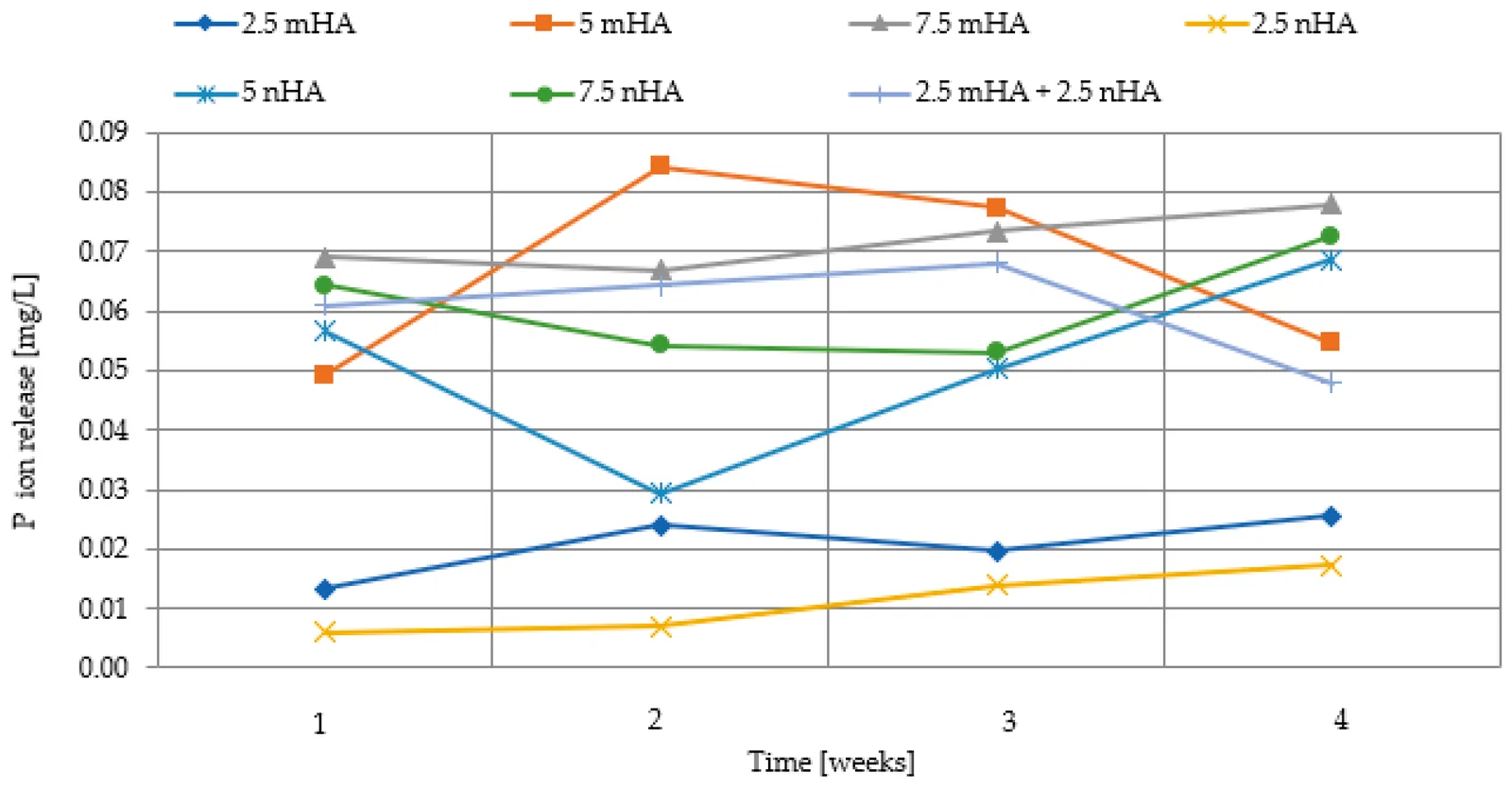
Dynamic of phosphorus (P) (177.495 nm) ion release via ICP-OES.

No quantifiable phosphorus ion release was observed in the control group. Similarly to calcium release patterns, ion release is dose-dependent and sustained over 4 weeks (Figure 6 and Table A6).

### 3.7. Scanning Electron Microscopy with Energy-Dispersive X-Ray Spectroscopy

**Figure 7:**
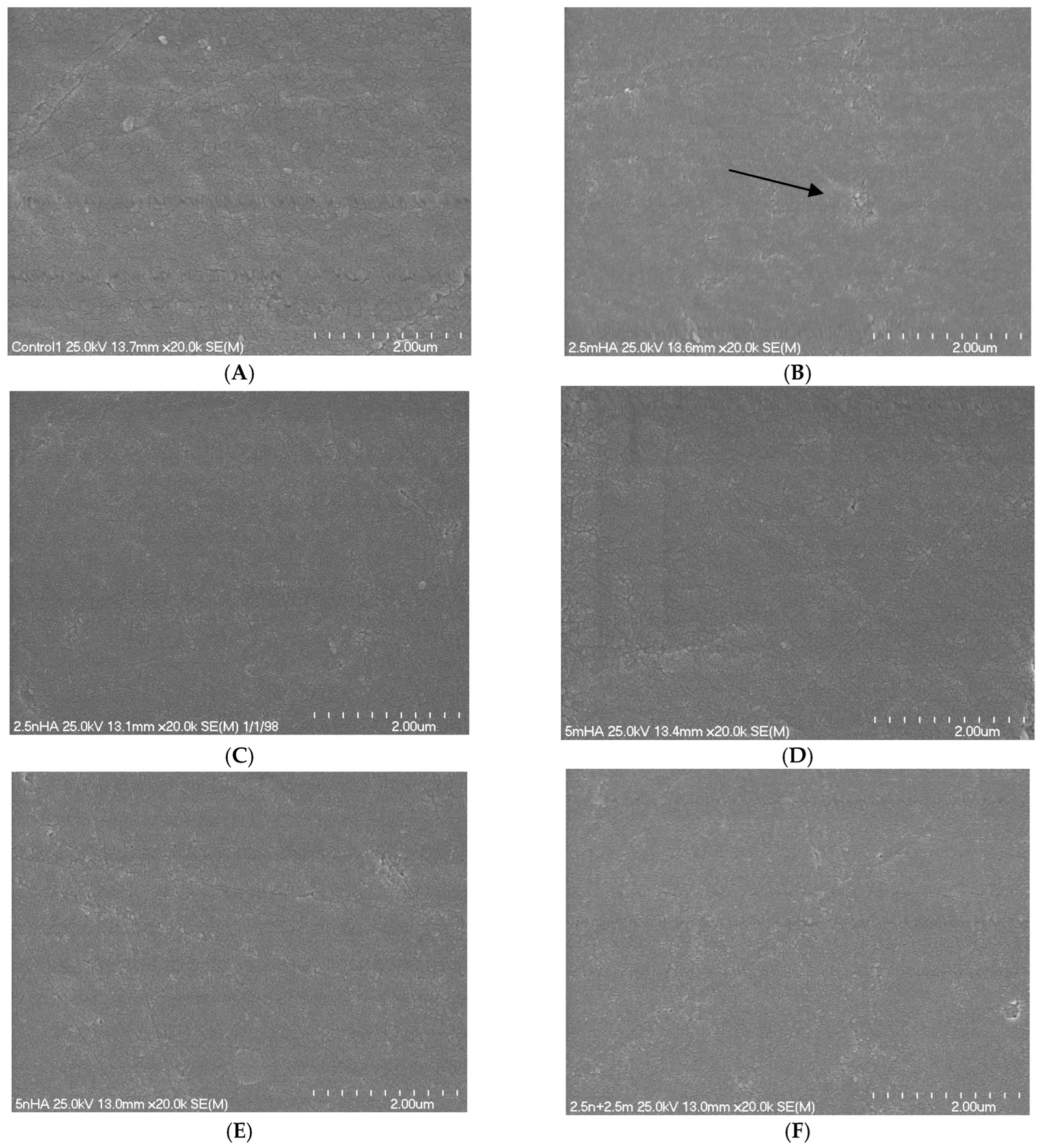

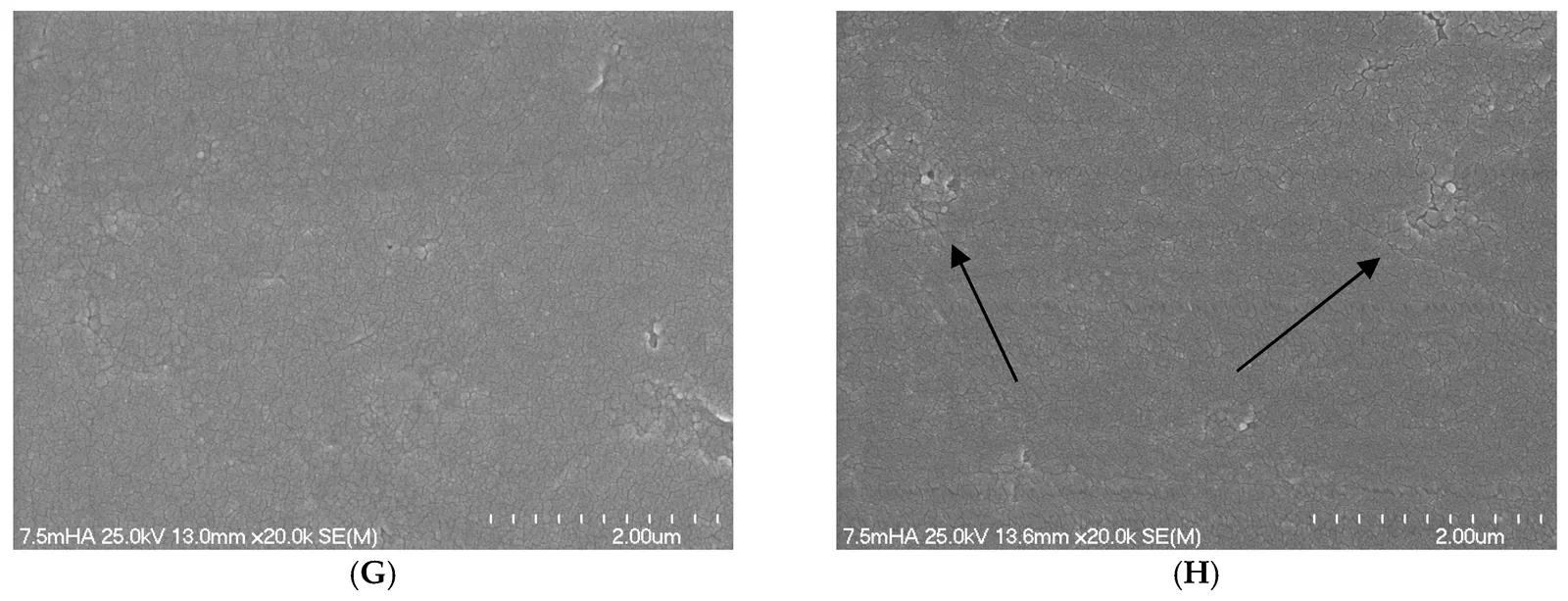
SEM micrographs of experimental materials (magnification 20,000×). (**A**) control group. (**B**) 2.5 wt.% mHA. (**C**) 2.5 wt.% nHA. (**D**) 5 wt.% mHA. (**E**) 5 wt.% nHA. (**F**) 5 wt.% hHA (2.5 mHA + 2.5 nHA). (**G**) 7.5 wt.% mHA. (**H**) 7.5 wt.% nHA.

SEM micrographs of the control group (Figure 7A) presented homogenous surface structure with regular distribution of composite phases and few irregularities. Material modified with 2.5 wt.% mHA demonstrated a less homogenous structure with several clusters likely attributed to uneven distribution of filler particles (arrow in Figure 7B). Material modified with 2.5 wt.% nHA was characterized by smooth, homogenous surface. In the materials modified with 5 wt.% HA, mHA produced the most even filler distribution and smooth surface, several clusters of unhomogenous distribution were observed in the material modified with 5 wt.% hHA. Uneven, unhomogenous surface morphology was also observed in both materials modified with 7.5 wt.% HA, more so for nHA (arrows at Figure 7H).

**Figure 8:**
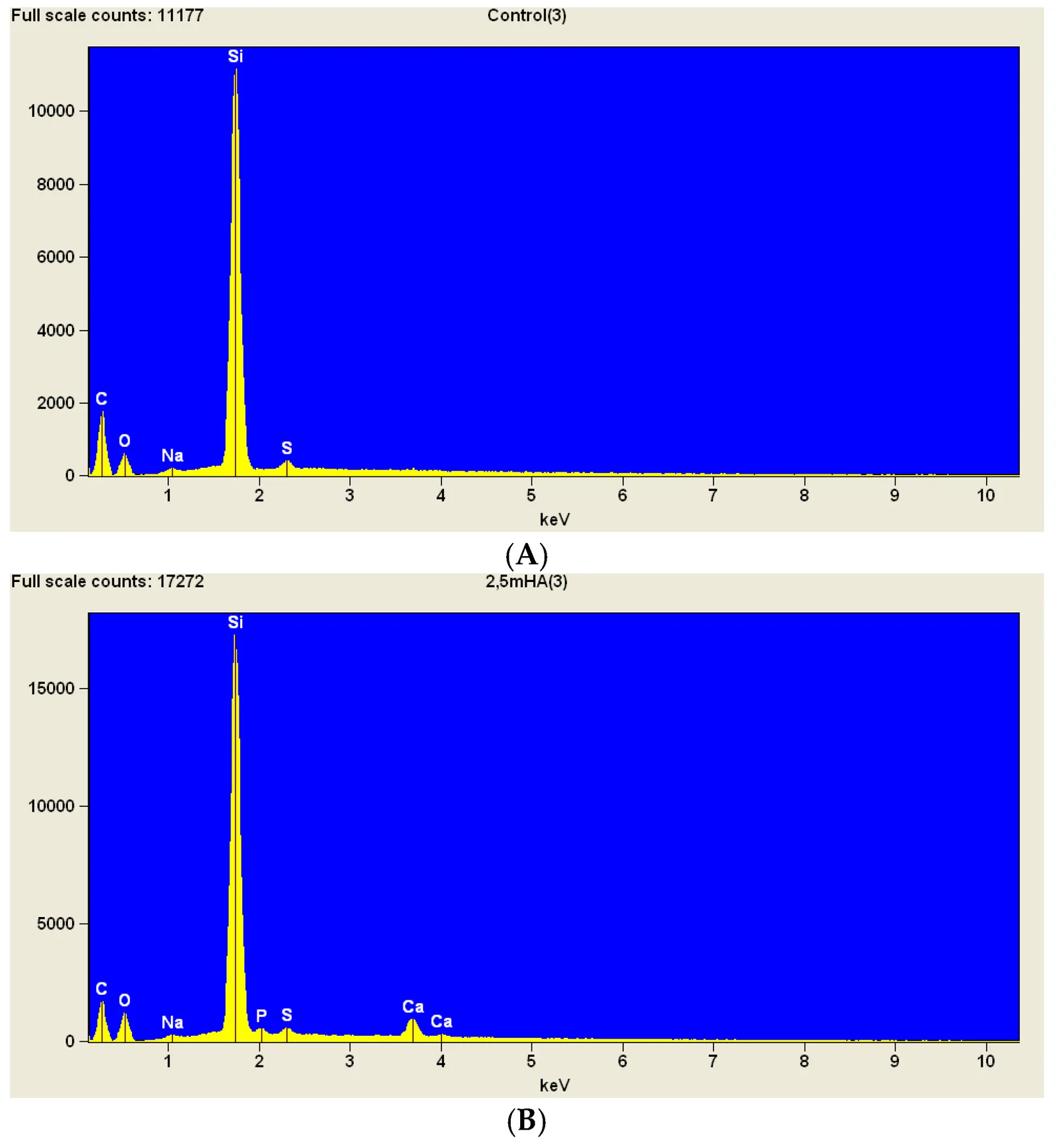

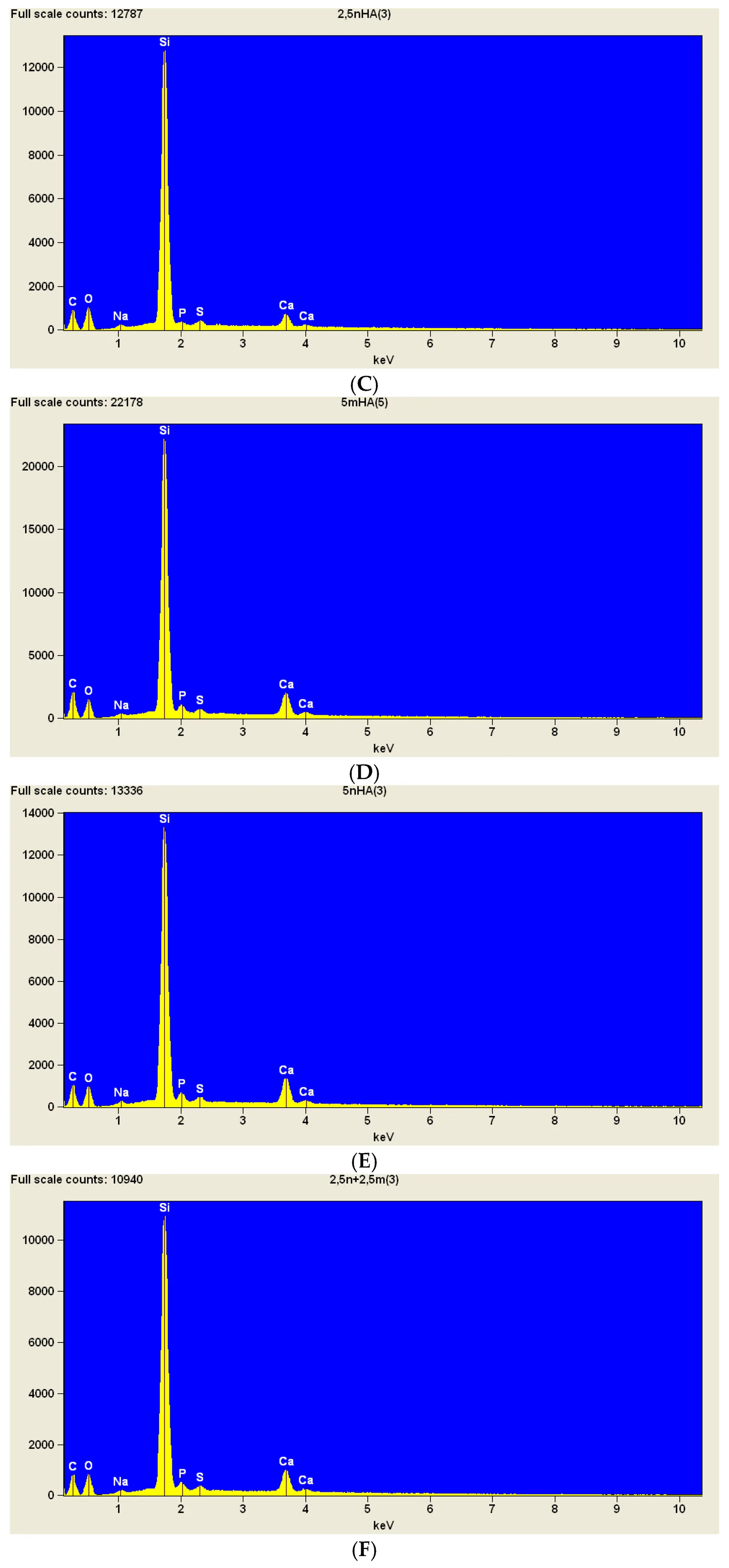

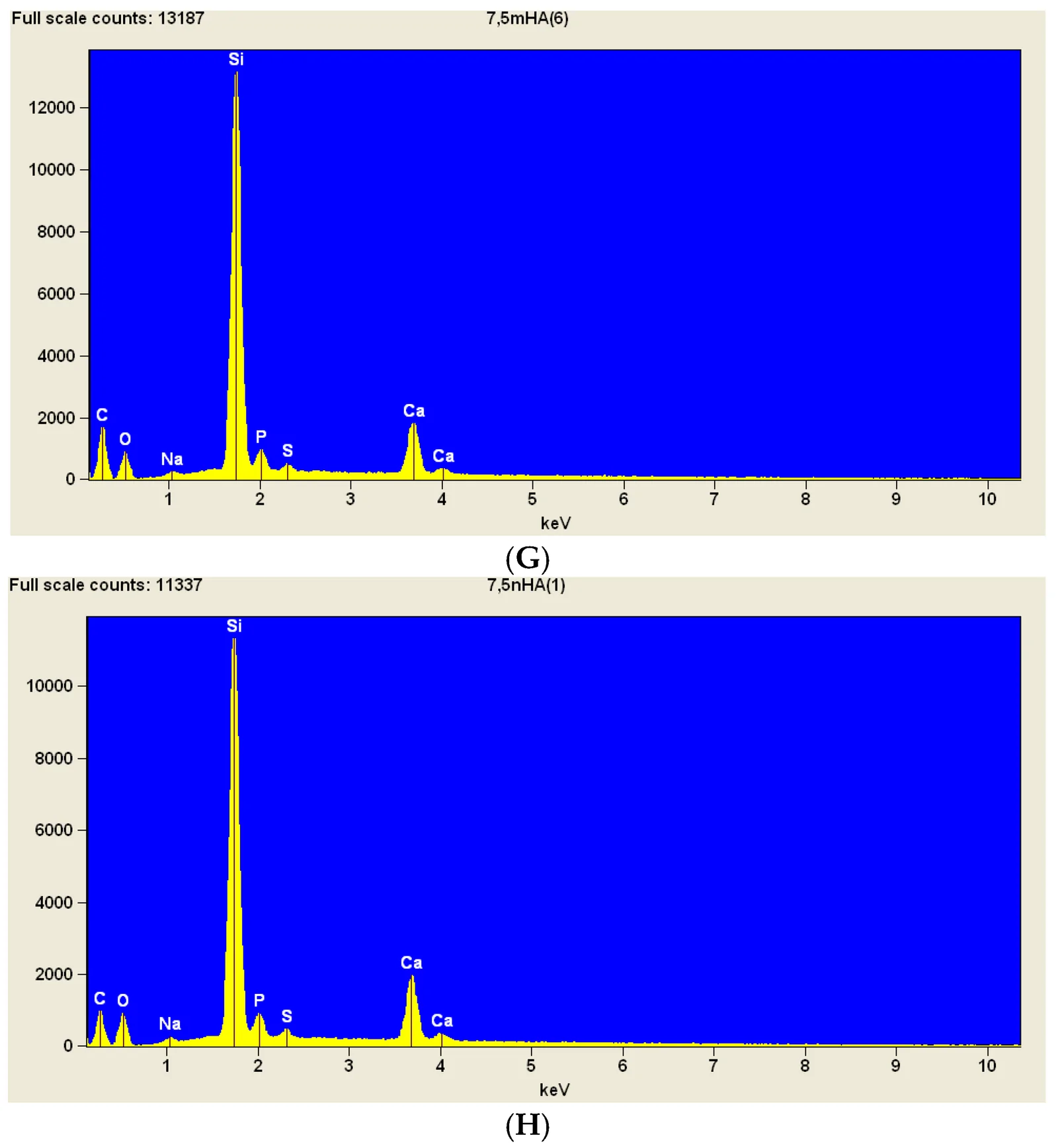
EDS spectra acquired at a magnification of 1000×, samples sputter-coated with a 108 Carbon/A coater; (**A**) control group, (**B**) 2.5 wt.% mHA, (**C**) 2.5 wt.% nHA, (**D**) 5 wt.% mHA, (**E**) 5 wt.% nHA, (**F**) 5 wt.% hHA (2.5 mHA + 2.5 nHA), (**G**) 7.5 wt.% mHA, (**H**) 7.5 wt.% nHA.

Elemental EDS analysis of the control showed the distribution of elements, with silicon being the most abundant. Carbon, oxygen, and sodium were present in quantifiable amounts (Figure 8A). No detectable traces of calcium or phosphorus were observed. Calcium and phosphorus were detected in all HA-modified RBCs. Their amount was increasing with the concentration of HA. In all experimental materials, calcium was more abundant than phosphorus.

**Figure 9:**
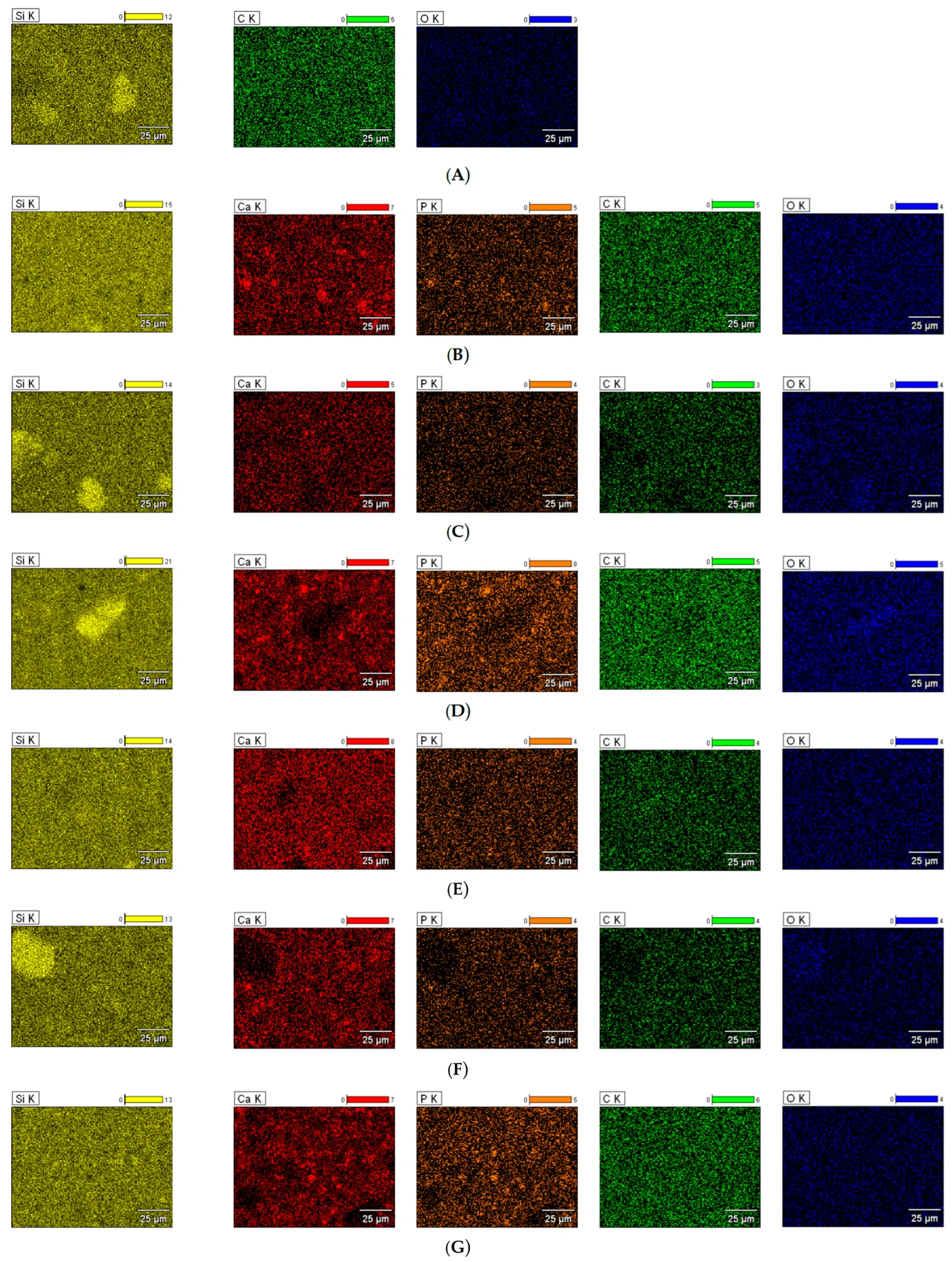

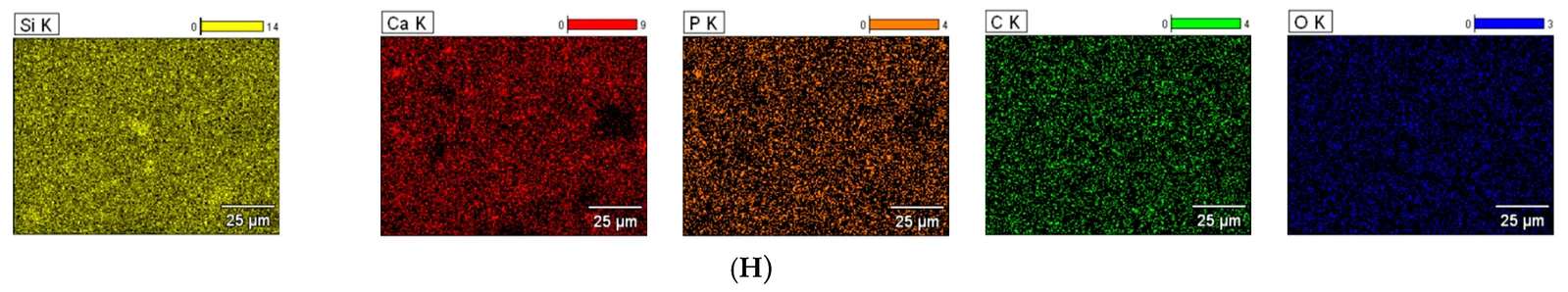
Elemental distribution maps of experimental materials(magnification 1000×), samples sputter-coated with a 108 Carbon/A coater; (**A**) control group, (**B**) 2.5 wt.% mHA, (**C**) 2.5 wt.% nHA, (**D**) 5 wt.% mHA, (**E**) 5 wt.% nHA, (**F**) 5 wt.% hHA (2.5 mHA + 2.5 nHA), (**G**) 7.5 wt.% mHA, (**H**) 7.5 wt.% nHA.

Material modified with 2.5 wt.% mHA demonstrated equally distributed silicon (Figure 9B). Calcium was detected and distributed relatively regularly, with some noticeable cluster of greater distribution in areas with lower distribution of silicon. However, there were also areas with a lower distribution of both silicon and calcium. Irregularly distributed phosphorus was also detected. Similar elemental composition was observed in material modified with 2.5 wt.% nHA and the distribution of the elements was less even, with clusters of silicon and calcium observed (Figure 9C). The pattern of distribution of calcium in areas with less silicon held true for most cases, but there were also areas with lower distribution of both calcium and silicon. Phosphorus distribution mirrored that of calcium. Material modified with 5 wt.% nHA presented a more regular elemental distribution (Figure 9E). Material modified with 5 wt.% hHA demonstrated uneven distribution of elements at this concentration with a notable Si cluster corresponding to lack of Ca and P (Figure 9F). All materials modified with 5 wt.% HA demonstrated less homogenous element distribution than those modified with 2.5 wt.% HA. The content of calcium and phosphorus was dose-dependent.

## 4. Discussion

Materials used for tissue replacement must have specific functional properties. While ISO 4049 requires only flexural strength to be tested, hardness and diametral tensile strength are also commonly tested [42].

### 4.1. Qualitative Observations

It has been noted that for both materials with the highest hydroxyapatite concentration the authors were unable to introduce the silica filler in originally intended concentrations. This might suggest that high HA concentrations might negatively influence the rheological properties of resin-based composite. Influence of this incorporation shortfall is revisited in Section 4.6. This limitation could be addressed by silanization of HA. However, while silanization of the filler particles could improve the incorporation and resultant mechanical properties of RBC [43,44], it would be likely to decrease its potential biofunctional behavior. Additionally, HEMA has been found to exhibit a tendency to interact with HA through its primary hydroxyl function, potentially facilitating distribution and bonding of the filler in the matrix [45]. While this proposed property provides rationale for inclusion of HEMA in the formulation, it should be noted that this monomer is rarely used in commercially available dental composite. It might increase water sorption and plasticize the polymer network [46]. Further consideration into the trade-off of including HEMA in the formulation is warranted.

### 4.2. Hardness

Hardness can be defined as the quantitative measure of resistance of a solid object to local deformation. Hardness values result from a defined measurement procedure and thus cannot be considered as an intrinsic material property. Indentation tests have been in use for well over a century. The principle behind them is the behavior of elastic or plastic materials in which the intender leaves an indentation behind after it is withdrawn [42]. Measurement of this property is necessary to determine the resistance of restoration to forces generated during mastication. Understanding this property is crucial, since the materials aim to mimic the tissues they are designed to replace. For enamel, the average hardness level is 274 HV, around 4.2 times higher than that of dentin [13,47].

The results of this study indicate that modification with HA increases the Vickers hardness of RBC, but this effect is non-monotonic. This result is more noticeable for nHA-modified materials. This effect could be explained by suboptimal incorporation of filler particles, as explained in Section 4.6. Based on current literature, RBCs used in dentistry are characterized by minimum HV values ranging between 40 and 50 [13,48]. Commercial composites will typically reach HV levels from 70 to above 110 [13,41]. Materials tested in this study do not seem to meet the HV requirements for commercial RBC, but it should be noted that commercial composites have typically higher overall filler load, which strongly improves their HV [13,43,49]. Flowable materials and experimental composites are characterized by HV values closer to those in the present study [50,51,52]. Additionally, silanization of filler particles has been found to markedly increase the HV [43]. Zhang and Darvell tested RBC modified with silanized HA whiskers (116 ± 23 μm × 102 ± 32 μm) and aggregated HA nanopowder (5–20 μm). Their study showed significant increase in HV with increasing filler loading from 22 HV at 0 vol.% HA to over 60 and 80 HV for 30 vol.% particles and whiskers, respectively [45]. Lung et al. researched Bis-GMA/TEGDMA (60/40 wt.%) RBC modified with silanized HA particles and unsilanized HA particles (35–125 nm) at 10 and 30 wt.%. They measured Knoop hardness, finding that incorporation of HA improved surface hardness, with 30 wt.% silanized HA filler producing the most satisfactory results [44]. Calabrese et al. compared experimental RBC composed of HA whiskers (35 nm × 200 nm) introduced into Bis-GMA/TEGDMA (49.5/49.5 wt.%) alongside silica particles, with constant 30 vol.% resin and HA taking up 0, 20, 40, 60, 80, and 100 vol.% of the filler. The Vickers hardness of the control was 58, then increased to 77 HV at 20 vol.% HA (accounting for 11.6 wt.% HA) and decreased successively at higher concentrations, down to 53 HV at 52.5 wt.% HA [53]. Mirajkar et al. compared the Vickers hardness of RBC based on Bis-GMA/TEGDMA with filler composed of silanized nanohydroxyapatite, zirconia and glass in three concentrations (group 1 nHA/zirconia/glass at 24.7/22.1/14.5 vol %, group 2 at 26.7/26/17 vol % and group 3 at 32/27/19 vol % respectively). The Vickers hardness of these materials was 42.16 ± 4.57, 61.90 ± 5.35 and 81.76 ± 4.56, respectively [31]. Yadav and Gnagwar modified RBC containing Bis-GMA/HEMA/TEGDMA (60/19.5/19.5 wt.%) with nano-hydroxyapatite particles treated with 3-methacryloxypropyl trimethoxysilane (MPTS) or 3-aminopropyl triethoxysilane (APTES) at 0, 4, 8, and 12 wt.%, finding that Vickers hardness increased with dose of HA filler, in contrast to this manuscript, reaching maximum values at 12 wt.% (34.10 HV for MPTS-treated nHA and 33.31 HV for APTES-treated nHA) [54]. Differences in methodology and type of HA should be noted when comparing these studies.

### 4.3. Strength

Strength of the material is not its inherent property, but rather a function of sample composition, specimen preparation, and geometry, as well as the chosen testing method and represents resistance to catastrophic fracture. In dental material science, it is difficult to replicate the typical loading conditions that materials will meet in the oral cavity. Simple axial loading is rarely encountered due to the three-dimensional movements of the masticatory system and complex tooth anatomy. Tensile loading is considered the most appropriate. However, flexure testing is also commonly used [42].

### 4.4. Diametral Tensile Strength

The results of this study imply that modification with HA generally improves the diametral tensile strength of resin-based composites, most notably for 5 wt.% formulations. This result is slightly more observable for mHA-modified RBC. The standard DTS established by the American Dental Association for commercial RBC is no less than 24 MPa [41]. All materials produced in this study meet this criterion. Materials with low DTS are recommended for lining materials and for non-chewing surfaces [13].

### 4.5. Flexural Strength

In light of this study, modification with HA has mixed results on the flexural strength of the composite. SEM-EDS evidences filler particle aggregation and formation of voids, which might explain the non-monotonic strength pattern, as discussed in Section 4.6. nHA-modified materials yield slightly better results than mHA-modified ones. Minimum FS for RBC used on occlusal surfaces in the posterior region (Class I/Class II) is 80 MPa, and for less loaded surfaces (Class V/deciduous teeth/lining), it is 50 MPa, generally ranging from 60 to 180 MPa in commonly used RBC [13,29,41,43,54,55]. Only RBC modified with 5 wt.% nHA meets both criteria, but all experimental materials meet the second criterion. In line with the results of this study, Zhang and Darvell also obtained different behavior in regard to FS for HA-modified materials. There was no significant change for whisker HA filler, with FS levels above 100 MPa for all groups. However, aggregated HA nanopowder resulted in a decrease in FS with increasing filler concentration from 100 MPa at 0 vol.% to 25 MPa at 30 vol.% [45]. Ai et al. performed a three-point bending test on RBC composed of silver nanoparticles-laden HA nanowires (≈200 nm) introduced into Bis-GMA/TEGDMA (50/50 wt.%) matrix at 0, 4, 6, 8, and 10 wt.%. The control group displayed FS of 106.1 MPa. HA nanowires were found to significantly increase FS at 6 and 8 wt.% by 20–30 MPa and decrease above that level [56]. Calabrese et al. found a progressive decrease in FS with increasing HA concentrations [53]. Aydinoglu et al. used 7 wt.% rod-shaped, biomimetic HA (mean particle size < 300 nm) to modify RBC composed of Bis-GMA, HEMA, UDMA and TEDGMA (4.5/10/10/4.5 wt.%) and silanized SiO_2_ with Si/Zr nanoclusters (31.5/31.5 wt.%). Their materials displayed FS above 80 MPa, but addition of HA decreased FS in comparison to control group [4]. Yadav and Gangwar observed decrease in FS with increasing nHA content, reaching minimum values at 12 wt.% (80.72 MPa for MPTS-treated HA and 83.63 MPa for APTES-treated HA) [54]. Zhang et al. prepared RBC modified with fluorinated urchin-like hydroxyapatite (FUHA) and urchin-like hydroxyapatite (UHA) at 30 or 50 wt.% introduced into resin containing Bis-GMA/TEGDMA/UDMA at 39.6/29.7/29.7 wt.% and compared them with commercial RBC Z350XT and BEAUTIFIL II. The highest FS (137.17 ± 4.53 MPa) was demonstrated by materials filled with 50 wt.% FUHA [57].

### 4.6. Summary of Mechanical Properties

As shown in Figure 1, Figure 2, Figure 3 and Figure 4, modification with HA tends to improve HV and DTS of experimental composite, but has mixed effects on FS, as discussed in Section 4.2, Section 4.4, and Section 4.5. The most likely explanation for the impact of HA modification on the basic mechanical properties of RBC would be the increase in overall filler content, leading to some improvement in tested qualities [43]. However, the highest concentrations of HA are not as effective as this explanation might imply. This would suggest that functional filler is not optimally incorporated into the matrix. Mechanical properties are influenced not only by the filler volume, but also matrix composition, filler shape and morphology, size and distribution, as well as the quality of interfacial bonding [45]. While it is true that increasing filler content generally improves the mechanical properties of the material, it is only true up to a certain volume and only if the requirements for the other factors are met as well. SEM-EDS, as discussed in Section 4.8, evidences filler particle aggregation and formation of voids, which might explain the non-monotonic strength pattern by creating weak points which fail under stress. Additionally, lack of HA filler functionalization could negatively influence mechanical performance via debonding under load. Silanization of HA filler might improve the mechanical properties of the material [44].

Summarized multi-criteria scoring was assigned to each group, as seen in Table 3. For each tested parameter, the group with the highest median value was assigned a score of 100%. The other groups were assigned scores relative to that value. An average of parameter scores with equal weighting was assigned to each group as an overall score. Groups were ranked according to their overall scores to determine the most mechanically optimal formulation. Taking these results into consideration, the modification of RBC with 5 wt.% HA is the optimal concentration with regard to manufacturing feasibility as well as mechanical properties, but the mechanical properties–biofunctional potential trade-off should be considered in future studies, as discussed in Section 4.9 and Section 4.10.

### 4.7. Inductively Coupled Plasma Optical Emission Spectrometry of Ion Release

Ion release results are explanatory in nature. ICP-OES provide evidence of a dose-dependent calcium and phosphorus ion release in HA-modified materials. This effect is sustained over the period of four weeks, indicating potential long-term calcium and phosphorus ion release. Biofunctional potential favors higher HA formulations, as presented in Table 4, but the material’s primary function cannot be impaired. Comparing these results with mechanical performance, formulations with 5 wt.% HA provide the best mechanical properties while exhibiting satisfactory potential biofunctional performance.

Buchwald et al. tested experimental RBC containing 50 wt.% Bis-GMA/TEGDMA resin in 3:2 ratio modified with 50 wt.% filler composed of HA particles (mean particle size 10 μm), either unmodified, silanized, modified with heptaisobutyltri((methacryloxypropyl) dimethylsiloxy) heptasilsesquioxane (HIB-met), or silanized and modified with HIB-met. Their findings demonstrated that all experimental RBC presented the ability to release calcium ions in deionized water, as detected by ion-selective electrode. However, this process would decrease over the span of 12 weeks, which the authors attribute to its diffusion-controlled mechanism. A stable plateau was observed in all groups after 5 weeks. In the initial period, the highest Ca^2+^ ion release was observed in RBC containing unmodified HA and silanized HA, corresponding to the solubility of RBC. After reaching the plateau, all examined materials presented similar Ca^2+^ ion release [58]. Balhuc et al. report that nanosized HA displays better bioactivity [59]. Jardim et al. investigated three RBCs modified with different concentrations of HA nanoparticles, finding a long-term Ca^2+^ and PO_4_^3−^ release [60]. A review by Vilela et al. notes that HA particles are relatively less soluble than other calcium orthophosphates, such as brushite, which may contribute to HA’s lower ion release in comparison. They also note that while functionalization of ion-releasing particles improves the mechanical performance of the material, it may limit its ion release [61]. Many studies test ion release from HA-modified RBCs in deionized water rather than physiological saline. Resende et al. modified RBC containing equal ratio of Bis-GMA/TEGDMA with 30 vol.% HA. Samples immersed in ultrapure water for 4 weeks were tested by ICP-OES, similarly to this study, showing a steady release of Ca^2+^ ions over time with cumulative ion release after 28 days at 64 μg/cm^2^ (7.0 ppm) [62]. Zhang et al. demonstrated that RBC modified with 50 wt.% UHA or FUHA immersed with water presented a pronounced calcium and phosphorus ion release after 7 days, followed by a gradual and steady release. UHA-modified RBC presented marginally higher ion release, likely caused by increased resistance to hydrolysis in fluoride-modified FUHA [57].

Summarized multi-criteria scoring was assigned to each group, as seen in Table 4. For each tested ion release, the averaged of median values across four measurement periods was calculated to represent sustained ion release score. Values < LOQ were normalized as zero, but it should be noted that this is an approximation. The group with the highest mean value was assigned a score of 100%. The other groups were assigned scores relative to that value. An average of parameter scores with equal weighting was assigned to each group as an overall score. Groups were ranked according to their overall scores to determine the most biofunctionally optimal formulation. Based on these results, 7.5 wt.% formulation yield superior results, but 5 wt.% formulation also provide satisfactory results.

### 4.8. Scanning Electron Microscopy with Energy-Dispersive X-Ray Spectroscopy

**Figure 10:**
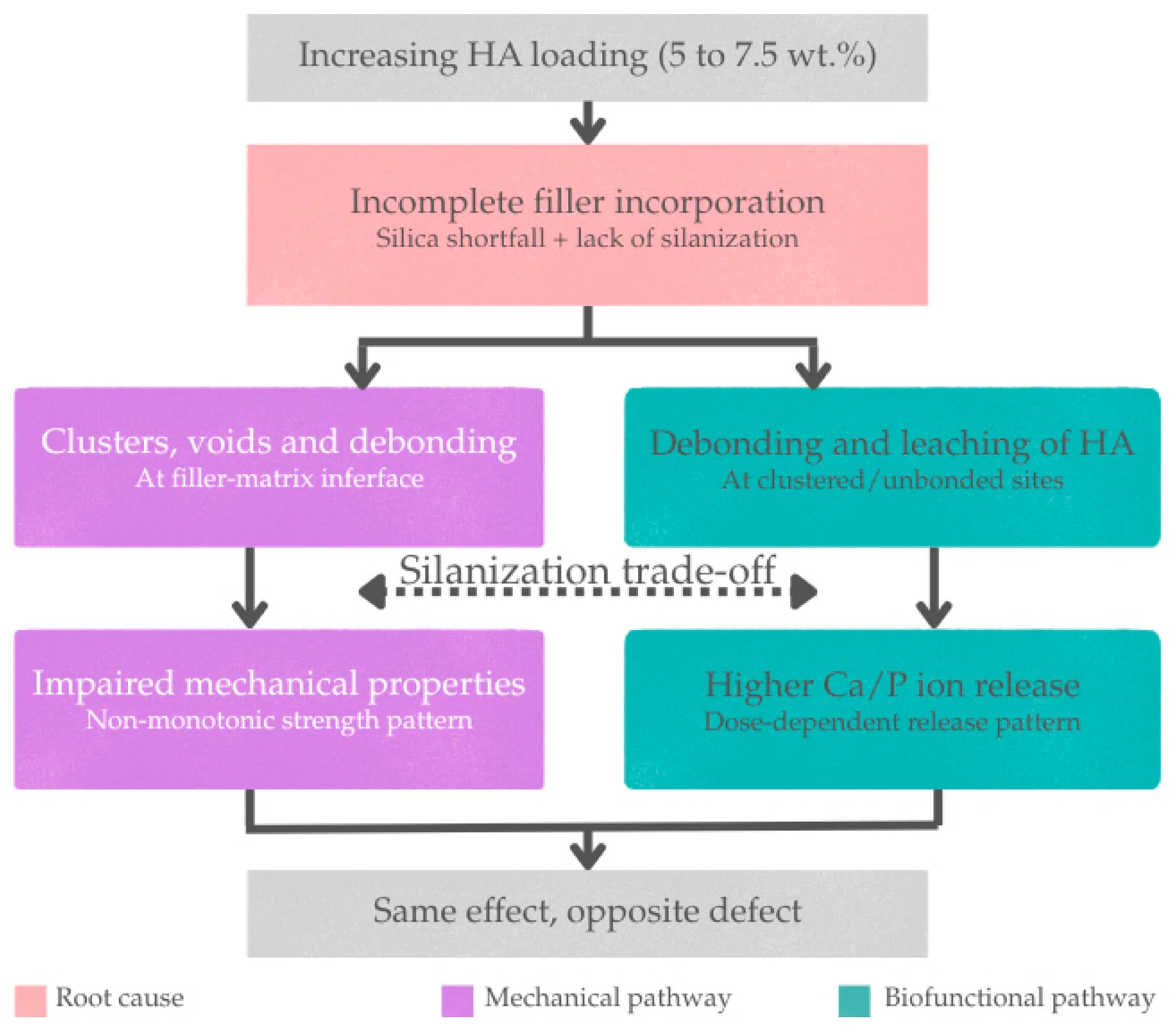
Schematic summary of the proposed relationship between incomplete silica incorporation at higher filler loadings and its divergent effects on mechanical performance and ion release.

There was no detectable amount of calcium or phosphorus in the control group during EDS analysis. The content of calcium and phosphorus increases with increasing HA concentrations. However, increasing the concentration of HA would negatively impact the homogenous distribution of the elements in the material, suggesting that the filler particles are not evenly distributed, providing physical grounding for non-monotonic strength pattern discussed in Section 4.6 and presented in Figure 10.

This effect is more noticeable for nHA-modified materials, suggesting nanoparticles are less suitable for introduction as a functional filler. Ai et al. observed that HA nanowires were equally distributed throughout the resin matrix at concentrations 4–8 wt.% with no major aggregates, in contrast to this study. Calcium was evenly dispersed at those concentrations. However, increasing the amount of HA nanowires to 10 wt.% resulted in the aggregation of calcium elements in some domains and visible bundles of HA nanowires [56]. Lung et al. analyzed materials modified with 10 and 30 wt.% silanized and unsilanized HA nanopowder under SEM, observing globular hydroxyapatite particles, and concluding that silanization of filler particles improved the properties of the material [44]. Resende et al. observed that materials modified with HA showed well-dispersed filler particles in the matrix, but less homogenous microstructure under SEM in comparison to control [62].

### 4.9. Summary of Biofunctional Potential

The findings of this study suggest that calcium and phosphorus ion release is dose-dependent and sustained over time. However, consistent with the scope defined in Section 1.1, this can be only considered as preliminary evidence of potential biofunctional behavior. Lack of HA filler silanization and its suboptimal distribution could influence the chemical mechanism of ion release. Specifically, lack of HA filler functionalization might facilitate leaching and detachment of HA particles into the environment. Additionally, suboptimal distribution and formation of HA clusters on the surface of the material could increase ion release. More defined understanding of the mechanism of biofunctional behavior and its duration are required by the FDI framework for bioactivity assessment, as discussed in Section 4.10.

Scores obtained for mechanical performance and biofunctional potential were used in Table 5 to assign an overall score as an average of scores obtained in Table 3 and Table 4. It should be noted that these scores are relative to the highest performing group in each category and that results supporting biofunctional potential are preliminary in nature. In conclusion, 5 wt.% formulations appear optimal, considering also manufacturing feasibility.

### 4.10. Methodological Limitations and Recommendations for Future Research

The difference between the intended filler content and the actual filler content presents a serious limitation of this study, as discussed in Section 4.1. The authors need to explicitly acknowledge that all comparisons are based on the actual, rather than nominal, material compositions. Incomplete filler incorporation, especially at higher concentrations, may have influenced mechanical performance and chemical behavior. Specifically, suboptimal incorporation of filler could result in formation of aggregation and voids, as well as debonding under load, as discussed in Section 4.6. It could also influence the materials chemical behavior by facilitating leaching and detachment of HA particles, as discussed in Section 4.9. Proposed mechanisms are presented in Figure 10. Surface functionalization of HA particles is an important gap to be covered by future studies.

This study did not include any aging protocols. Inclusion of assays such as thermocycling or water aging would provide more informed predictions on long-term behavior of modified materials and expected duration of biofunctional potential, as required by the FDI framework introduced in Section 1.1. Specifically, any changes in mechanical properties with aging and the mechanism of sustained ion release over time highly influence the potential clinical application of HA-modified materials. Performing SEM-EDS analysis after storage with appropriate aging protocols would additionally provide clinically relevant data in regard to surface-related properties of the materials.

Future studies regarding ion release should develop precise contamination control and handle the materials in media such as artificial saliva or simulated body fluid to imitate oral environment. pH controls and studying the effects of acidic solutions on ion release and stability of the composite would also provide more clinically relevant information.

**Figure 11:**
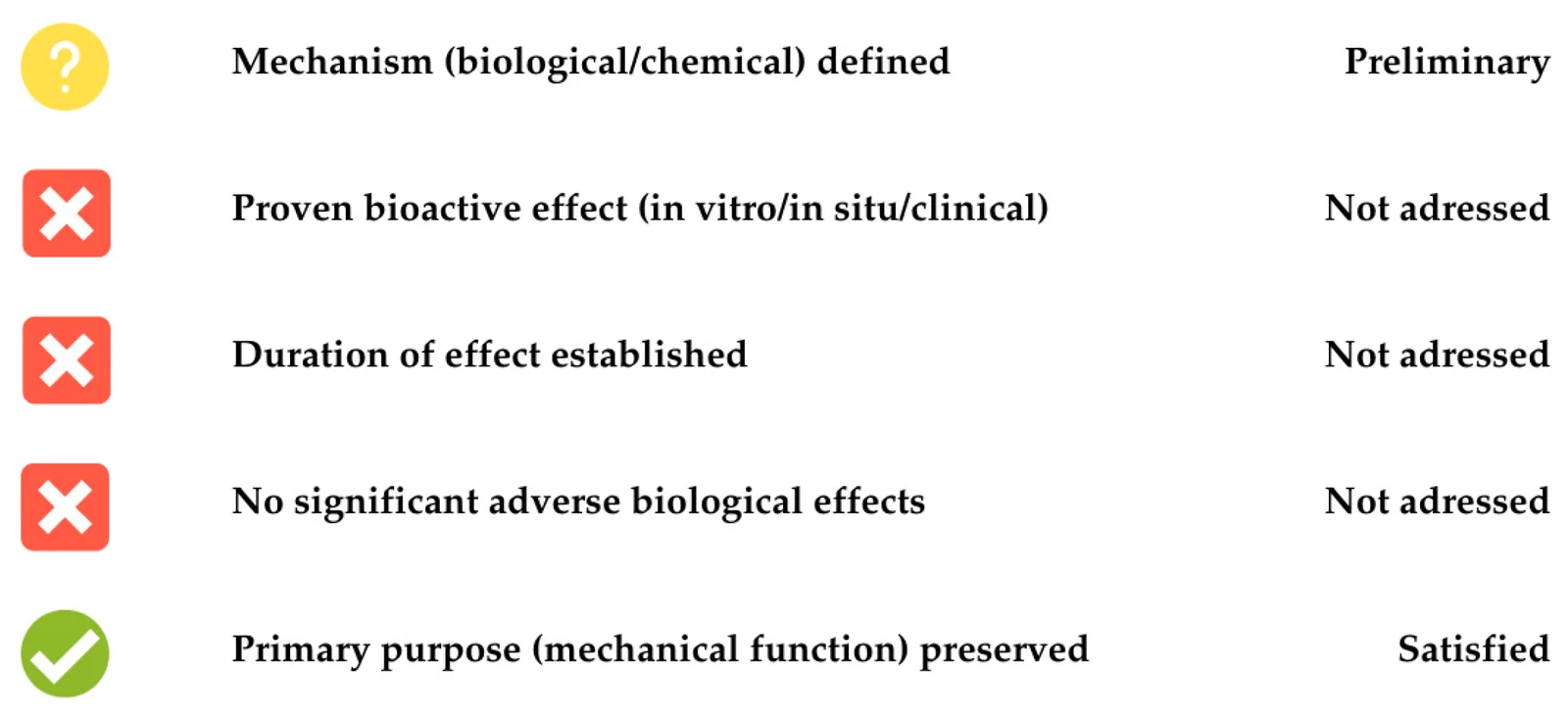
Status of FDI 2022 bioactivity framework criteria in this paper.

The tested materials meet the minimum standards for DTS and FS for commercial composites, but regarding HV, they are comparable to flowable materials. The experimental materials meet one of the FDI criteria for bioactive restorative dental materials—the primary function of the material is not impaired. They also provide evidence of calcium and phosphate ion release, which could be understood as preliminary evidence of biofunctional potential. The preliminary evidence of biofunctional potential encourages further research into the bioactivity of HA as a functional filler. However, it is important to underline that in vitro ion release data cannot be taken as primary evidence of bioactivity. Available data and literature indicate ion release is one of potential chemical mechanisms of bioactivity that HA-modified RBCs are capable. More comprehensive research designed to fully examine potential bioactivity of HA-modified RBC should be structured around the FDI framework for dental bioactive materials. The satisfaction of the FDI criteria within the scope of this study is presented in Figure 11.

## 5. Conclusions

Mindful of the limitations of this study highlighted above, it is possible to present the following key findings in regard to the properties of HA-modified resin-based composite:

- Modification with HA improves the Vickers hardness and diametral tensile strength of RBC, but has mixed effects on the flexural strength;
- The tested materials meet the minimum requirements for DTS and TPB for commercial composites, but with regard to HV, they require further improvement;
- There is dose-dependent calcium and phosphate ion release in materials modified with HA.

The results of this study partially reject the null hypothesis as posed in Section 1.2 in regard to mechanical properties and biofunctional potential. The experimental materials meet one of the FDI criteria for bioactive restorative dental materials—the primary function of the material is not impaired. There is preliminary evidence of chemical biofunctional potential via ion release in in vitro setting, as required by FDI criteria. This conclusion highlights the feasibility of research into the bioactivity of HA-modified composite designed to fully satisfy the FDI criteria for bioactive restorative dental materials. Further research is recommended to evaluate and clearly describe the mechanism of bioactivity of HA-modified composites and the duration of this effect. Materials should also be tested for significant adverse biological effects.

## Figures and Tables

**Table 1:** Proposed composition of experimental materials.

Group		Control	2.5 mHA	5 mHA	7.5 mHA	2.5 nHA	5 nHA	7.5 nHA	2.5 mHA + 2.5 nHA
Composition		wt.%	wt.%	wt.%	wt.%	wt.%	wt.%	wt.%	wt.%
Resin matrix		55.0	52.5	50.0	47.5	52.5	50.0	47.5	50.0
Filler	Silica	45.0	45.0	45.0	45.0	45.0	45.0	45.0	45.0
	mHA(2.5 μm)	0.0	2.5	5.0	7.5	0.0	0.0	0.0	2.5
	nHA (<200 nm)	0.0	0.0	0.0	0.0	2.5	5.0	7.5	2.5

**Table 2:** Obtained composition of experimental materials.

Group		Control	2.5 mHA	5.0 mHA	7.5 mHA	2.5 nHA	5.0 nHA	7.5 nHA	2.5 mHA + 2.5 nHA
Composition		wt.%	wt.%	wt.%	wt.%	wt.%	wt.%	wt.%	wt.%
Matrix	Bis-GMA 35, UDMA 35, TEGDMA 20, HEMA 10	55.2	52.6	50.3	48.7	52.5	50.1	49.4	50.1
Filler	Silica	44.8	44.9	44.7	43.5	44.9	44.9	42.8	44.9
	mHA (2.5 μm)	0.0	2.5	5.0	7.8	0.0	0.0	0.0	2.5
	nHA (<200 nm)	0.0	0.0	0.0	0.0	2.6	5.0	7.9	2.5

**Table 3:** Summary of mechanical properties relative to the highest performing group.

Group	HV Score [%]	DTS Score [%]	FS Score [%]	Ef Score [%]	Overall Score [%]
5 wt.% nHA	97	94	100	94	96
5 wt.% hHA	94	100	84	90	92
5 wt.% mHA	91	98	82	88	90
7.5 wt.% mHA	88	85	70	100	86
2.5 wt.% nHA	100	80	72	90	85
2.5 wt.% mHA	94	70	87	86	84
7.5 wt.% nHA	94	81	67	91	83
Control	88	78	68	89	81

**Table 4:** Summary of biofunctional potential relative to the highest performing group.

Group	Ca Ion Release Score [%]	P Ion Release Score [%]	Overall Score [%]
7.5 wt.% nHA	100	100	100
7.5 wt.% mHA	92	100	96
5 wt.% hHA	97	88	92
5 wt.% nHA	91	88	89
5 wt.% mHA	89	81	85
2.5 wt.% mHA	42	27	34
2.5 wt.% nHA	40	24	32
Control	31	0	15

**Table 5:** Overall mechanical behavior and biofunctional performance scores relative to the highest performing group.

Group	Overall Mechanical Score [%]	Overall Biofunctional Potential Score [%]	Overall Score [%]
5 wt.% nHA	96	89	92
5 wt.% hHA	92	92	92
7.5 wt.% nHA	83	100	91
7.5 wt.% mHA	86	96	91
5 wt.% mHA	90	85	87
2.5 wt.% mHA	84	34	59
2.5 wt.% nHA	85	32	58
Control	81	15	48

## Data Availability

The original contributions presented in this study are included in this article. Further inquiries can be directed to the corresponding author.

## Appendix A

**Table A1:** Vickers hardness [-] of experimental materials.

Group *n* = 9	Control	2.5 mHA	5.0 mHA	7.5 mHA	2.5 nHA	5.0 nHA	7.5 nHA	2.5 mHA + 2.5 nHA
Mean	30	31	31	30	33	33	32	32
Median	30	32	31	30	34	33	32	32
Minimum	29	28	29	29	30	32	30	31
Maximum	31	33	33	32	35	34	34	34
Standard deviation	1	2	1	1	2	1	1	1
Coefficient of variation	0.02	0.05	0.04	0.04	0.05	0.02	0.04	0.03

**Table A2:** Diametral tensile strength [MPa] of experimental materials.

Group *n* = 11	Control	2.5 mHA	5.0 mHA	7.5 mHA	2.5 nHA	5.0 nHA	7.5 nHA	2.5 mHA + 2.5 nHA
Mean	31.2	31.5	40.2	35.4	33.4	37.3	32.7	40.4
Median	32.2	28.9	40.2	35.0	33.0	38.5	33.1	41.0
Minimum	21.6	20.6	37.2	29.2	21.8	23.5	22.6	35.0
Maximum	41.4	42.0	43.9	42.3	44.4	44.6	39.5	45.8
Standard deviation	6.9	7.6	2.4	5.5	7.4	5.5	6.0	3.5
Coefficient of variation	0.22	0.24	0.06	0.16	0.22	0.15	0.19	0.09

**Table A3:** Flexural strength [MPa] of experimental materials.

Group *n* = 7	Control	2.5 mHA	5.0 mHA	7.5 mHA	2.5 nHA	5.0 nHA	7.5 nHA	2.5 mHA + 2.5 nHA
Mean	66.1	77.4	67.2	56.4	62.4	95.1	60.4	76.5
Median	62.2	80.1	74.9	64.0	65.9	91.5	61.1	76.6
Minimum	33.8	67.9	47.3	30.1	37.6	76.7	37.4	63.9
Maximum	102.0	82.9	78.6	67.5	79.9	127.0	75.0	94.7
Standard deviation	20.2	5.6	12.1	15.2	16.2	16.9	14.0	10.2
Coefficient of variation	0.31	0.07	0.18	0.27	0.26	0.18	0.23	0.13

**Table A4:** Flexural modulus [MPa] of experimental materials.

Group *n* = 7	Control	2.5 mHA	5.0 mHA	7.5 mHA	2.5 nHA	5.0 nHA	7.5 nHA	2.5 mHA + 2.5 nHA
Mean	4591	4196	4297	4783	4340	4821	4513	4379
Median	4330	4170	4280	4860	4380	4570	4430	4420
Minimum	4090	4000	3920	4400	4100	4340	4260	4120
Maximum	6490	4360	4540	4980	4530	6370	4930	4760
Standard deviation	843	128	203	187	161	705	245	245
Coefficient of variation	0.18	0.03	0.05	0.04	0.04	0.15	0.05	0.06

**Table A5:** ICP-OES analysis of Ca (393.366 nm) ion release [mg/L].

Group *n* = 3		Control	2.5 mHA	5 mHA	7.5 mHA	2.5 nHA	5 nHA	7.5 nHA	2.5 mHA + 2.5 nHA
Time									
1 week	Mean	0.04	0.05	0.13	0.16	0.05	0.14	0.16	0.15
	Median	0.04	0.05	0.11	0.13	0.06	0.16	0.19	0.11
	Minimum	0.03	0.04	0.07	0.13	0.03	0.05	0.11	0.08
	Maximum	0.05	0.07	0.20	0.22	0.06	0.20	0.19	0.27
	Standard deviation	0.01	0.01	0.07	0.05	0.02	0.08	0.04	0.11
	Coefficient of variation	0.22	0.29	0.54	0.33	0.37	0.55	0.28	0.69
2 weeks	Mean	0.03	0.08	0.20	0.15	0.04	0.09	0.13	0.17
	Median	0.03	0.06	0.24	0.15	0.04	0.10	0.13	0.17
	Minimum	0.03	0.05	0.11	0.13	0.03	0.08	0.11	0.11
	Maximum	0.03	0.14	0.26	0.18	0.06	0.10	0.15	0.22
	Standard deviation	0.00	0.05	0.08	0.02	0.02	0.01	0.02	0.05
	Coefficient ofvariation	0.08	0.64	0.40	0.15	0.38	0.16	0.15	0.33
3 weeks	Mean	0.09	0.09	0.18	0.19	0.07	0.13	0.13	0.20
	Median	0.09	0.08	0.13	0.18	0.07	0.15	0.14	0.22
	Minimum	0.07	0.06	0.10	0.17	0.05	0.08	0.11	0.15
	Maximum	0.10	0.11	0.32	0.22	0.09	0.17	0.15	0.24
	Standard deviation	0.01	0.02	0.12	0.02	0.02	0.05	0.02	0.05
	Coefficient of variation	0.15	0.29	0.65	0.13	0.28	0.35	0.17	0.23
4 weeks	Mean	0.05	0.10	0.13	0.18	0.08	0.17	0.19	0.15
	Median	0.05	0.09	0.12	0.16	0.10	0.20	0.21	0.15
	Minimum	0.03	0.06	0.10	0.16	0.06	0.10	0.13	0.11
	Maximum	0.06	0.13	0.16	0.22	0.10	0.21	0.23	0.18
	Standard deviation	0.01	0.04	0.03	0.03	0.02	0.06	0.05	0.04
	Coefficient of variation	0.30	0.38	0.24	0.19	0.23	0.35	0.28	0.25

**Table A6:** ICP-OES analysis of P (177.495 nm) ion release via ICP-OES [mg/L].

Group *n* = 3		Control	2.5 mHA	5 mHA	7.5 mHA	2.5 nHA	5 nHA	7.5 nHA	2.5 mHA + 2.5 nHA
Time									
1 week	Mean	<LOQ	0.01	0.05	0.07	0.01	0.06	0.06	0.06
	Median	<LOQ	0.01	0.03	0.06	0.01	0.07	0.06	0.04
	Minimum	<LOQ	0.01	0.03	0.05	0.01	0.02	0.05	0.03
	Maximum	<LOQ	0.02	0.09	0.10	0.01	0.08	0.09	0.12
	Standard deviation	No data	0.01	0.04	0.03	0.00	0.03	0.02	0.05
	Coefficient of variation	No data	0.38	0.77	0.39	0.17	0.60	0.30	0.82
2 weeks	Mean	<LOQ	0.02	0.08	0.07	0.01	0.03	0.05	0.06
	Median	<LOQ	0.02	0.09	0.07	0.01	0.03	0.06	0.06
	Minimum	<LOQ	0.01	0.04	0.06	0.01	0.02	0.05	0.04
	Maximum	<LOQ	0.04	0.12	0.07	0.01	0.04	0.06	0.09
	Standard deviation	No data	0.01	0.04	0.00	0.00	0.01	0.01	0.03
	Coefficient of variation	No data	0.55	0.49	0.07	0.25	0.24	0.15	0.41
3 weeks	Mean	<LOQ	0.02	0.08	0.07	0.01	0.05	0.05	0.07
	Median	<LOQ	0.02	0.04	0.06	0.01	0.05	0.05	0.08
	Minimum	<LOQ	0.02	0.04	0.05	0.01	0.03	0.05	0.03
	Maximum	<LOQ	0.03	0.16	0.10	0.02	0.07	0.06	0.09
	Standard deviation	No data	0.01	0.07	0.03	0.00	0.02	0.01	0.03
	Coefficient of variation	No data	0.37	0.87	0.36	0.31	0.37	0.16	0.49
4 weeks	Mean	<LOQ	0.03	0.05	0.08	0.02	0.07	0.07	0.05
	Median	<LOQ	0.02	0.05	0.07	0.02	0.08	0.09	0.05
	Minimum	<LOQ	0.01	0.04	0.07	0.01	0.03	0.04	0.03
	Maximum	<LOQ	0.04	0.08	0.10	0.03	0.10	0.09	0.07
	Standard deviation	No data	0.02	0.02	0.02	0.01	0.03	0.03	0.02
	Coefficient of variation	No data	0.61	0.41	0.22	0.55	0.48	0.40	0.41
